# Localization Before Exploration: A Proposed Risk-Stratified Outpatient Framework for Occult Cutaneous Foreign Bodies

**DOI:** 10.7759/cureus.113730

**Published:** 2026-07-31

**Authors:** Wen-Guo Chen, Wen-Tsao Ho

**Affiliations:** 1 Dermatology, Ho Wen-Tsao Skin Clinic, New Taipei City, TWN

**Keywords:** cutaneous foreign body, foreign body localization, outpatient surgery, procedural safety, radiography, shared decision-making, soft-tissue foreign body, ultrasonography

## Abstract

Suspected superficial foreign bodies create pressure for immediate removal, although the target may no longer be visible or palpable at examination. In this report, an occult cutaneous foreign body is a suspected object confined to the skin or superficial subcutis that cannot be directly visualized or reliably palpated after initial assessment. Repeated probing in this setting can enlarge the wound, distort tissue planes, and endanger adjacent structures without assuring retrieval. This technical report presents a proposed localization-first outpatient framework for clinically stable patients. It separates retention hazard from exploration hazard, defines a localization threshold before limited intervention, and introduces a pre-agreed procedural stop rule. Radiography is prioritized when radiopaque material is plausible, whereas high-frequency ultrasonography is used for radiolucent material, uncertain depth, or proximity to important structures; a negative ultrasound examination is not treated as definitive exclusion. When immediate intervention is not justified, active observation comprises wound care, tetanus assessment, scheduled reassessment, and explicit return precautions. Once a lesion becomes clinically or sonographically localized, targeted removal may be considered by limited incision, image guidance, or carefully selected punch or elliptical excision. The supporting evidence is a focused narrative synthesis rather than a systematic review, and the two-hazard matrix, localization threshold, follow-up intervals, and stop rule are proposed operational components rather than validated recommendations. No comparative data establish that this framework improves retrieval, safety, or tissue preservation; prospective evaluation is required.

## Introduction

Small splinters, thorns, glass fragments, and other superficial foreign bodies are common reasons for outpatient and emergency consultation, although their exact clinical burden is difficult to quantify because uncomplicated injuries are frequently treated outside formal care and published series are anatomically and methodologically heterogeneous. In this report, an occult cutaneous foreign body is a suspected object confined to the skin or superficial subcutis that cannot be directly visualized or reliably palpated after the initial examination. This definition excludes deeper penetrating injuries and suspected involvement of joints, tendons, named neurovascular structures, body cavities, or other sites that require emergency or specialist pathways.

Removal is usually straightforward when an object is visible, palpable, or accurately localized. The more difficult problem arises when the history remains convincing, but the object cannot be seen or felt. In this circumstance, the puncture site may be mistaken for the target, and persistence may be mistaken for technical thoroughness. Repeated exploration can enlarge the wound, create bleeding that further obscures the field, disrupt normal tissue planes, injure a tendon or neurovascular structure, and still fail to retrieve the object [1]. Immediate removal is not the only defensible response to uncertainty. In a cohort of hand and wrist injuries, most foreign bodies that were left in place remained asymptomatic, and only a small minority required later removal [2]. This observation does not justify indiscriminate retention, particularly for organic material, infection, functional compromise, or high-risk anatomy; it shows that the possibility of retention is not, by itself, an indication for blind exploration.

Published literature largely addresses detection, retained-object sequelae, or retrieval techniques, but offers limited operational guidance on how an outpatient clinician should compare the hazard of retention with the hazard of exploration, decide when localization is adequate, or stop an unsuccessful attempt. This report therefore proposes a reproducible localization-first framework for clinically stable patients with suspected cutaneous or superficial subcutaneous foreign bodies. Its objectives are to define clinical scope, classify retention and exploration hazards independently, specify a threshold for limited intervention, and describe escalation, observation, and stop-rule pathways. The framework is a decision aid derived from published evidence and clinical reasoning, not a prospectively validated guideline.

## Technical report

Evidence basis and status of recommendations

The supporting literature was assembled as a focused narrative synthesis of clinically relevant studies addressing radiographic and ultrasonographic detection, outcomes of retained foreign bodies, inflammatory complications, and image-guided removal. This was not a systematic review: no prespecified protocol, duplicate screening, or formal certainty assessment was performed, and selection bias is possible. Statements about diagnostic performance, complications, and procedural outcomes are therefore tied to the cited evidence, whereas the two-hazard matrix, localization threshold, follow-up intervals, and stop rule are author-proposed operational components based on clinical reasoning. These components should not be interpreted as validated recommendations and require prospective evaluation.

Defining the clinical scope before attempting removal

The first task is not to choose an instrument but to decide whether the patient belongs in an office-based pathway. The clinician should document the mechanism, suspected material, time since injury, prior removal attempts, pain trajectory, contamination, immunocompromising conditions, neurovascular findings, range of motion, and the relationship of the puncture to joints, tendons, nail units, and named neurovascular structures. Progressive pain, spreading erythema, purulence, fever, neurologic deficit, vascular compromise, restricted tendon excursion, suspected joint penetration, or deep puncture should trigger prompt imaging, surgical consultation, or emergency referral rather than limited office exploration.

Material matters because retention risk is not uniform. Dry, inert, small fragments may remain clinically silent, whereas wood, thorns, plant material, and heavily contaminated objects may provoke inflammation, infection, or granuloma formation [1]. A high retention hazard is therefore an indication for timely localization and definitive management, not permission to search blindly.

Separating retention hazard from exploration hazard

Two independent questions should be answered. Retention hazard asks what may happen if the object remains. Exploration hazard asks what may happen if the clinician continues searching now. Retention hazard increases with reactive or contaminated material, infection, worsening symptoms, functional impairment, migration potential, and proximity to a joint, tendon, nerve, or vessel. Exploration hazard increases when the object is invisible and impalpable, depth or orientation is uncertain, normal anatomy cannot be distinguished, there have been previous failed attempts, or the proposed wound would be disproportionate to the expected benefit.

**Figure 1:**
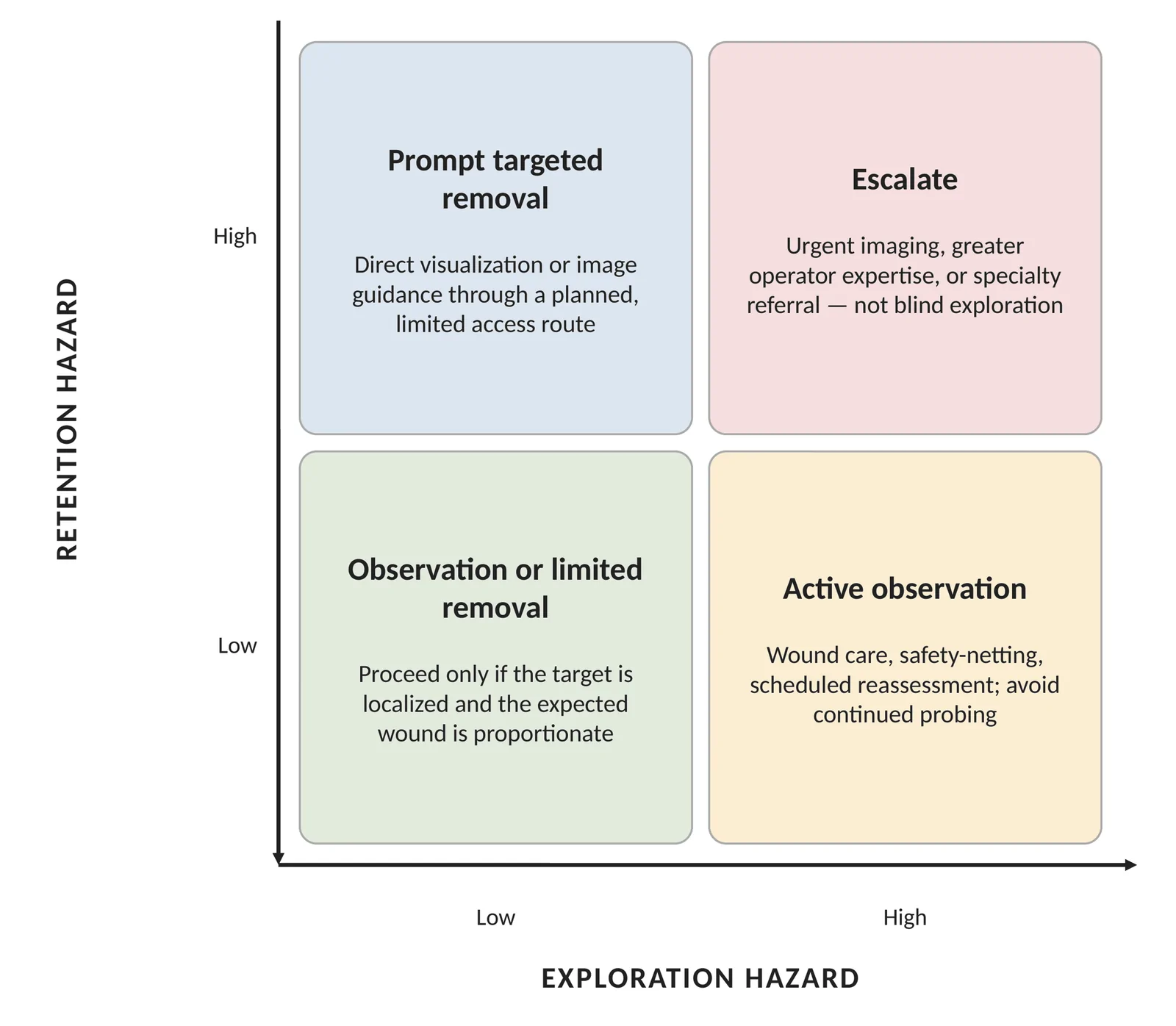
Two-hazard decision matrix for suspected occult cutaneous foreign bodies. Retention hazard (harm if the object remains) and exploration hazard (harm of searching now) are assessed independently, yielding four dispositions: observation or limited removal (both low), active observation (retention low, exploration high), prompt targeted removal (retention high, exploration low), and escalation to imaging, greater expertise, or referral (both high). Image created by authors using Microsoft PowerPoint (Microsoft Corporation, Redmond, WA).

This two-hazard model prevents a common logical error: using high retention hazard to justify high-risk blind exploration. Four dispositions follow. When both hazards are low, the clinician may discuss observation or limited removal if the target is localized and the expected wound is proportionate. When retention hazard is low, but exploration hazard is high, active observation with safety-netting is usually preferable to continued probing. When retention hazard is high, but exploration hazard is low, prompt targeted removal is reasonable. When both hazards are high, the correct response is escalation of localization, imaging, operator expertise, or specialty referral-not blind exploration (Figure 1).

Applying a localization threshold

Limited intervention should proceed only when a localization threshold has been met. In this protocol, the threshold requires a credible target, an estimate of depth and long-axis orientation, an acceptable understanding of adjacent important structures, and a planned access route that allows direct visualization or continuous image guidance while limiting tissue sacrifice. The final element is procedural discipline: the operator must be able to state in advance when the attempt will stop. An entry puncture alone does not satisfy the threshold because skin and soft tissue can shift relative to the retained object.

Clinical localization may be sufficient when a superficial fragment is directly visible or distinctly palpable, and its relation to important anatomy is clear. When any component remains uncertain, imaging should precede further manipulation. This reframes imaging from a test used after failed exploration to a means of determining whether exploration is justified at all.

Choosing imaging according to material and anatomy

Plain radiography is appropriate when glass, metal, stone, or another radiopaque material is plausible. Experimental data show excellent radiographic detection of radiopaque materials but poor detection of wood, plastic, and plant spines [3]. High-frequency ultrasonography is useful for radiolucent material, superficial localization, estimation of depth and orientation, detection of surrounding inflammatory change, and assessment of proximity to tendons, vessels, and nerves [4-7].

Ultrasonography is operator-dependent and should not be used as a stand-alone rule-out test. Reported performance varies substantially across models, operators, and clinical settings; pooled evidence supports high specificity but only moderate sensitivity [3,8]. A positive, anatomically coherent examination can support targeted removal, but a negative study should lead to reassessment of pretest probability, material, depth, timing, and operator expertise. Persistent high-risk symptoms despite nondiagnostic imaging warrant referral or additional imaging rather than reassurance by test result alone.

Using a pre-agreed stop rule

Before local anesthesia or incision, the clinician should explain that the objective is limited, anatomically controlled retrieval rather than exploration without a boundary. The attempt stops if the target is no longer directly visualized or reliably palpated, the dissection deviates from the predicted tract, bleeding or pain prevents safe identification, important anatomy becomes uncertain, the required wound exceeds the consented plan, or the object is not retrieved within the planned limited attempt.

A stop rule protects both patient and operator from escalation driven by sunk cost or pressure to produce an immediate result. Stopping is not a procedural failure when the localization assumptions have become invalid. It is the point at which the safest next procedure changes from exploration to relocalization, image-guided removal, planned excision, or specialist referral.

Defining active observation as treatment

Observation is appropriate only after high-risk features have been actively excluded and the patient can return reliably. The wound should receive standard cleansing and dressing, and tetanus prophylaxis should be assessed according to current local recommendations. The clinician should document the unresolved diagnostic possibility, the reason blind exploration is being avoided, and the agreed escalation plan. Return precautions should include increasing pain, erythema, warmth, swelling, drainage, fever, numbness, color change, restricted movement, or a newly palpable lesion.

Follow-up timing should reflect uncertainty rather than follow a fixed universal interval. As a pragmatic component of this proposed protocol, wounds with uncertain material, contamination, evolving symptoms, or prior probing are reviewed within 48-72 hours. Stable, low-risk wounds may be reassessed within 7-14 days, with earlier review for any warning feature. These intervals are operational suggestions and have not been validated in comparative trials.

Performing delayed targeted removal only after localization

Some retained superficial objects later become easier to localize because a focal papule, nodule, sinus, or sonographic inflammatory halo develops. Chronic inflammation and granuloma formation around retained material are well described [7,9]. Delayed localization can therefore change the benefit-to-harm ratio of removal, but it should not be assumed to occur in every patient. Persistent symptoms without localization require renewed imaging or referral.

When a lesion is superficial, discrete, and safely separated from important structures, targeted removal can be performed through a limited incision, ultrasound guidance, or a small punch or elliptical excision selected by a clinician competent in cutaneous surgery. The target should be marked before local anesthetic distorts the lesion. The excised tissue and wound bed should be inspected, and the removed material should be correlated with the expected object. Ultrasound-guided approaches can achieve high retrieval success while limiting tissue disruption when appropriate expertise is available [10-12]. If the object is not found during the planned attempt, serial blind punches or progressively wider probing should not follow. The localization model should be reconsidered, and the patient should be escalated to repeat imaging, image-guided retrieval, planned surgery, or specialist care.

**Figure 2:**
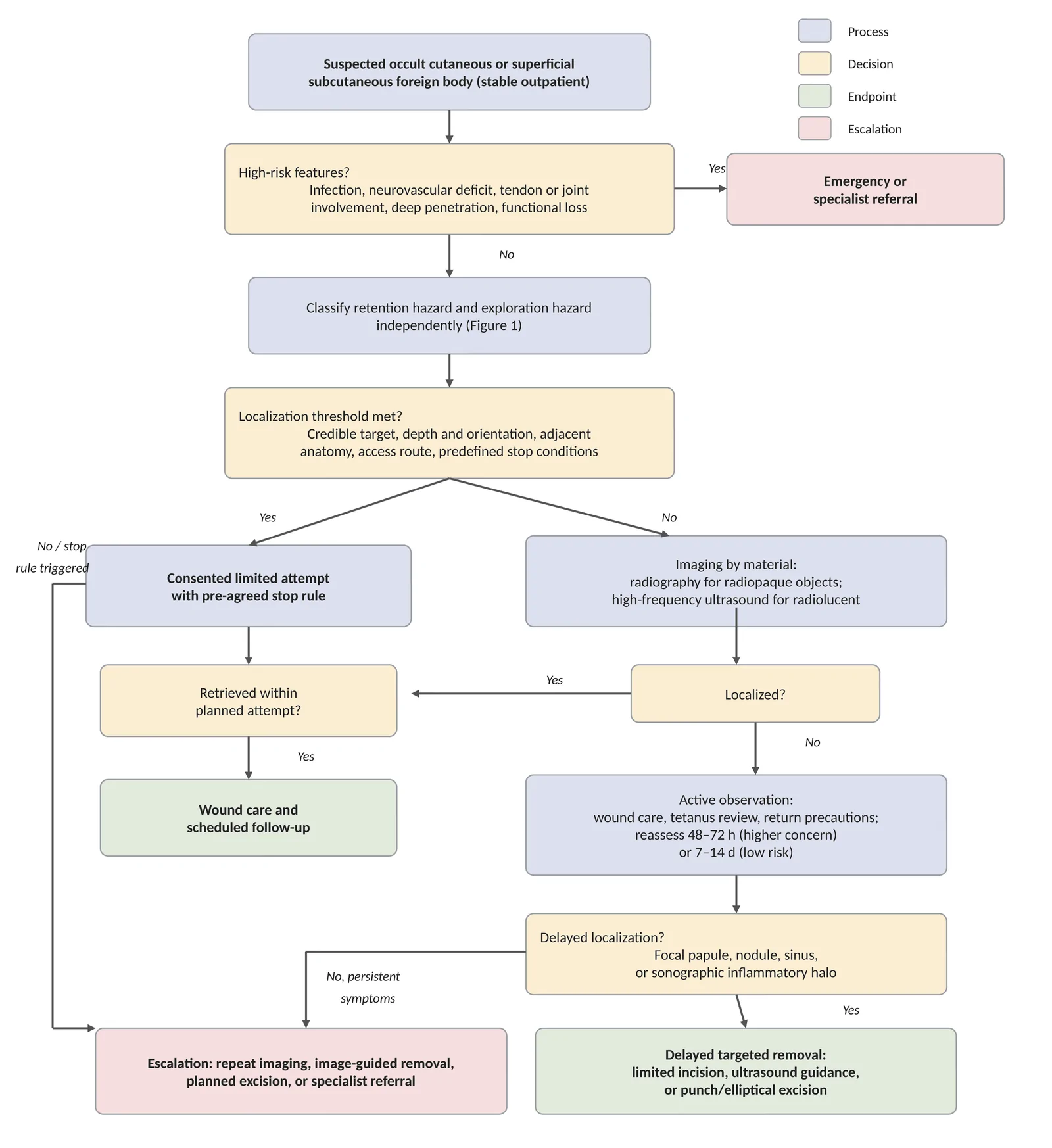
Localization-first outpatient protocol for occult cutaneous foreign bodies. The protocol screens for high-risk features, classifies retention and exploration hazards independently, and permits limited intervention only when a localization threshold is met. A consented attempt is bounded by a pre-agreed stop rule; if the object is not retrieved, the patient is escalated rather than subjected to progressively wider probing. When the threshold is not met, imaging is selected by material (radiography for radiopaque objects, high-frequency ultrasound for radiolucent objects), followed by active observation and delayed targeted removal only after clinical or sonographic localization. Image created by authors using Microsoft PowerPoint (Microsoft Corporation, Redmond, WA). US, ultrasound

The complete operational sequence is therefore: define whether office management is appropriate; classify retention and exploration hazards independently; require a localization threshold; choose imaging according to material and anatomy; undertake only a consented limited attempt with a stop rule; use structured active observation for low-risk unresolved cases; and perform delayed targeted removal only after clinical or sonographic localization (Figure 2). At every stage, infection, neurovascular change, functional loss, deep penetration, discordant imaging, high-risk anatomy, loss of the target, or failure of the planned attempt triggers escalation rather than progressively wider blind probing.

## Discussion

The proposed framework is localization-first rather than uniformly conservative. Its purpose is not to delay removal when delay may be harmful; it is to prevent diagnostic uncertainty from being converted into unbounded exploration. The two-hazard model clarifies why retention hazard and exploration hazard should be considered separately. Organic material near a tendon may create high retention hazard, but uncertainty about its location also creates high exploration hazard. The proposed response is urgent localization and appropriate referral, not immediate blind incision.

The localization threshold converts a general instruction to proceed cautiously into an auditable decision. Before intervention, the clinician identifies the target, depth, orientation, adjacent anatomy, access route, and stop conditions. This definition is compatible with direct clinical localization, radiography, or ultrasonography and does not privilege a single modality. Evidence for ultrasound is interpreted asymmetrically: a positive, anatomically coherent result may support targeted intervention, whereas a negative result cannot reliably exclude a retained object because sensitivity varies by material, size, operator, and setting [3,4,8].

Observation in this framework is an active management state rather than abandonment. The hand and wrist cohort reported by Potini et al. supports the possibility that selected retained foreign bodies can remain asymptomatic [2], but its retrospective design, anatomic focus, and reliance on subsequent encounters limit generalizability. Observation is therefore proposed only for stable, low-risk patients who can return reliably, with explicit warning signs and a defined route back to imaging or removal. Greater caution is warranted for plant-derived material, infection, progressive symptoms, functional impairment, and high-risk anatomy.

The evidentiary status of the framework's components differs. Statements about radiographic and ultrasonographic detection, retained-object sequelae, inflammatory change, and image-guided retrieval derive from the cited diagnostic, observational, and procedural studies. In contrast, the two-hazard matrix, localization threshold, suggested follow-up intervals, and pre-agreed stop rule are author-proposed operational components based on clinical reasoning. The stop rule addresses escalation after sunk effort, but no comparative study has shown that it reduces tissue injury or improves outcomes. These distinctions are important because the framework organizes existing evidence; it does not generate original patient-level or experimental results.

This report has important limitations. It is a focused narrative synthesis rather than a systematic review; there was no prespecified search protocol, duplicate screening, or formal certainty assessment, and literature-selection bias is possible. The cited studies are heterogeneous in material, anatomy, operator expertise, and outcome definitions. The proposed components have not been prospectively validated or compared with immediate exploration or usual care. The framework does not replace clinician judgment, imaging expertise, or specialty referral and is not intended for deep penetrating trauma, emergency conditions, or anatomically complex injuries. Some foreign bodies remain occult despite appropriate imaging, and delayed localization may never occur.

Prospective evaluation should measure successful retrieval, wound length or tissue volume removed, procedure duration, pain, infection, neurovascular or tendon injury, unplanned referral, repeat procedures, time to definitive management, scar outcome, and patient-reported confidence. A pragmatic comparison with usual care could test whether a localization-first pathway changes repeated blind attempts without increasing complications from retention. Until such data are available, the framework should be used only as a transparent decision aid with shared decision-making and explicit acknowledgment of uncertainty; improved safety, tissue preservation, or superiority should not be inferred.

## Conclusions

An unlocalized suspected cutaneous foreign body is a diagnostic problem, not an automatic indication for wider exploration. This report proposes a localization-first outpatient framework in which retention hazard and exploration hazard are considered independently, limited intervention is undertaken only after a defined localization threshold is met, and each attempt is bounded by a pre-agreed stop rule. Stable, low-risk cases may be managed with active observation and structured follow-up, whereas infection, reactive material, functional impairment, deep penetration, or high-risk anatomy warrants timely imaging, image-guided removal, or specialist referral. The framework is intended to make decisions to explore, observe, or escalate explicit and auditable, but it has not been prospectively validated and should not be interpreted as superior to usual care. Comparative studies are needed to determine its effects on retrieval, tissue injury, complications, time to definitive management, and patient-reported outcomes.
